# The Biomechanics of Musculoskeletal Tissues during Activities of Daily Living: Dynamic Assessment Using Quantitative Transmission-Mode Ultrasound Techniques

**DOI:** 10.3390/healthcare12131254

**Published:** 2024-06-24

**Authors:** Scott C. Wearing, Sue L. Hooper, Christian M. Langton, Michael Keiner, Thomas Horstmann, Nathalie Crevier-Denoix, Philippe Pourcelot

**Affiliations:** 1 School of Medicine and Health, Technical University of Munich, 80992 Munich, Bavaria, Germany; 2 School of Health, University of the Sunshine Coast, Sippy Downs, QLD 4556, Australia; 3 Griffith Centre of Rehabilitation Engineering, Griffith University, Southport, QLD 4222, Australia; 4 Department of Exercise and Training Science, German University of Health and Sport, 85737 Ismaning, Bavaria, Germany; 5 INRAE, BPLC Unit, Ecole Nationale Vétérinaire d’Alfort, 94700 Maisons-Alfort, France

**Keywords:** quantitative ultrasound, speed of sound, broadband ultrasound attenuation, connective tissue properties

## Abstract

The measurement of musculoskeletal tissue properties and loading patterns during physical activity is important for understanding the adaptation mechanisms of tissues such as bone, tendon, and muscle tissues, particularly with injury and repair. Although the properties and loading of these connective tissues have been quantified using direct measurement techniques, these methods are highly invasive and often prevent or interfere with normal activity patterns. Indirect biomechanical methods, such as estimates based on electromyography, ultrasound, and inverse dynamics, are used more widely but are known to yield different parameter values than direct measurements. Through a series of literature searches of electronic databases, including Pubmed, Embase, Web of Science, and IEEE Explore, this paper reviews current methods used for the *in vivo* measurement of human musculoskeletal tissue and describes the operating principals, application, and emerging research findings gained from the use of quantitative transmission-mode ultrasound measurement techniques to non-invasively characterize human bone, tendon, and muscle properties at rest and during activities of daily living. In contrast to standard ultrasound imaging approaches, these techniques assess the interaction between ultrasound compression waves and connective tissues to provide quantifiable parameters associated with the structure, instantaneous elastic modulus, and density of tissues. By taking advantage of the physical relationship between the axial velocity of ultrasound compression waves and the instantaneous modulus of the propagation material, these techniques can also be used to estimate the *in vivo* loading environment of relatively superficial soft connective tissues during sports and activities of daily living. This paper highlights key findings from clinical studies in which quantitative transmission-mode ultrasound has been used to measure the properties and loading of bone, tendon, and muscle tissue during common physical activities in healthy and pathological populations.

## 1. Introduction

Musculoskeletal disorders, including injuries of bone, tendon, and muscle tissue, affect more than 1.71 billion people worldwide [1]. The loss of bone mass associated with osteoporosis, for instance, is estimated to affect more than 500 million adults worldwide, with approximately nine million reportedly suffering an osteoporosis-related bone fracture each year, equating to an osteoporotic fracture every three seconds [2,3]. Similarly, the gradual loss of skeletal muscle mass and strength, characterizing sarcopenia, is conservatively estimated to affect as much as 10% of healthy older adults worldwide [4], while tendon disorders purportedly represent the primary musculoskeletal complaint for which a patient seeks medical attention [5]. Moreover, musculoskeletal disorders are a leading cause of chronic pain and protracted disability [6]. In 2020, musculoskeletal disorders were estimated to be the second-highest global cause of non-fatal disability [7]. Not surprisingly, musculoskeletal conditions are also the highest contributor to the global need for rehabilitation, with more than two-thirds of all adults worldwide in need of rehabilitative care [1].

The measurement of the mechanical properties of connective tissues and their change with physical loading is critical for understanding the cellular behavior of tissues, their normal ‘homeostatic’ tissue functionality, as well as the development of musculoskeletal disorders and their rehabilitation after injury [8]. The conceptual biomechanical model of the “mechanostat“, first proposed by Harold Frost in the 1980s, is arguably among the most significant contributions to connective tissue research to date [9,10,11]. The model states that connective tissues, including bone, tendon, and muscle tissues, respond to habitual loading and that any change in the loading environment leads to the structural adaptation of tissue architecture. The model suggests the presence of a physiological feedback system, which is able to adjust tissue mass and structure according to the loads experienced. Cells play a pivotal role in the model, acting both as sensors as well as drivers of extracellular matrix synthesis and degradation. The mechanostat is typically modeled as a feedback algorithm using a set-point criterion based on a particular mechanical quantity, such as strain or strain rate. Although the precise set-point criterion for many musculoskeletal tissues, including bone, tendon, and muscle tissues, is the source of ongoing debate [12,13,14,15,16], these mechanical thresholds (set points), determine whether material is added to or lost from the tissue. Different muscles, tendons, and bones are thought to respond differently to increases or decreases in loading depending on the sensitivity of their specific mechanostat [17,18]. It is unclear why and how the different connective tissue structures respond to complex loading stimuli, which comprise numerous different parameters including strain magnitude, frequency, and rest intervals (among others). When mechanical stimuli fall below a certain mechanical set point, cell apoptosis occurs followed by tissue resorption. Conversely, when mechanical loading remains within certain set points, cells remain viable and no tissue is lost (i.e., homeostasis). With a greater than customary mechanical stimulus, cells release anabolic factors, resulting in tissue formation. With excessive mechanical stimuli, the accumulation of micro-damage is thought to exceed tissue formation, resulting in injury [14,19]. Hence, understanding tissue properties and the physiological loading governing the mechano-sensitization and desensitization of individual musculoskeletal tissue structures is essential for our ability to promote positive musculoskeletal adaptation, prevent injury, hasten its recovery after injury, and enhance performance.

The primary aims of this narrative review, therefore, were threefold. The first aim was to review conventional methods currently used to quantify the mechanical properties of human musculoskeletal tissues, such as bone, tendon, and muscle tissues, *in vivo*, as well as to highlight the strengths and limitations of each approach. The second aim was to broadly outline current transmission-mode ultrasound techniques that have been specifically developed to non-invasively quantify the mechanical properties of human musculoskeletal tissues, including a brief overview of their operating principles and key measurement parameters that are commonly used. The third aim was to review important findings from *in vivo* applications of each technique in quantifying the mechanical properties and biomechanical loading of bone, tendon, and muscle tissues, both at rest and during activities of daily living in clinical and non-clinical populations. We also highlight new and emerging applications before concluding with a brief precis of future directions for the development and potential applications of transmission-mode techniques to evaluate the human musculoskeletal system.

To meet the aims of this review, we conducted a series of broad literature searches of a variety of electronic databases, including Pubmed, Embase, Scopus, Web of Science, and IEEE Explore, between 30 June 2023 and 1 May 2024. Keyword searches were carried out for each section of this review and included, but were not limited to, the following terms, used in varying combinations with and without the use of wild cards, truncation, and Boolean operators: skeletal, musculoskeletal, connective tissue, bone, osseous, trabecular, cortical, tendon, muscle, quantitative ultrasound, transverse transmission, through transmission, axial transmission, pulse echo, ultrasound, speed of sound, attenuation, osteopenia, osteoporosis, muscle wasting, sarcopenia, cachexia, tendinopathy, tendon rupture, tissue properties, biomechanics, viscoelasticity, modulus, bone mineral density, apparent density, apparent density, Poisson’s, and mechanobiology. No restrictions were placed on the publication date or type; hence textbooks, narrative and systematic review articles, meta-analyses, and original articles including, prospective and retrospective cohort studies, clinical trials, and observational studies, were included. A full-text review of sources was undertaken with respect to the hierarchy of the evidence and was limited to texts published in English or German languages. Additional sources were identified by scrutinizing the reference lists of published studies. In total, more than 800 peer-reviewed publications were retrieved and appraised, with 372 references directly included in the final review.

## 2. Conventional Methods for Characterizing Musculoskeletal Tissue Properties *In Vivo*

### 2.1. Bone

Dual-energy X-ray absorptiometry (DXA) is considered the current “gold-standard” method for assessing bone status *in vivo*. The technique quantifies the transmission and attenuation of two low-dose X-ray beams of separate energy, which are variably absorbed by hard and soft connective tissues, to provide a measure of areal bone mineral density (aBMD: g/cm^2^); which is used as a surrogate measure of bone strength [20,21,22]. Although DXA is the most widely used technique for evaluating osteoporosis and fracture risk in adults, its use in quantifying bone properties has several limitations [23]. In particular, as the technique is reliant on a two-dimensional (2-D) projection of skeletal tissues, it is unable to provide reliable quantitative information regarding true (i.e., 3-D) volumetric bone mineral density (vBMD: g/cm^3^) [24,25]. Moreover, normative data for aBMD have typically been reported only for common trabecular fracture sites, i.e., lumbar spine and hip [26], whereas 80% of osteoporosis-related fractures are estimated to involve the cortical bone [27]. Indeed, more than half of low-trauma fractures are reportedly undetected by DXA when the osteoporotic threshold defined by the World Health Organization (T-score = −2.5) is used [28], raising concerns regarding the sensitivity of DXA in detecting osteoporosis-related pathology [29,30]. In addition, the use of DXA in specific populations, such as children and adolescents, presents short-comings as DXA-based estimates of bone mineral density are highly dependent on bone morphology, body size, pubertal staging, and skeletal maturity [31]. Although high-resolution peripheral quantitative computed tomography (HR-pQCT) overcomes the 2-D limitations associated with DXA to provide 3-D information about cortical bone at peripheral skeletal sites [32,33], it currently provides insufficient spatial resolution to accurately evaluate intra-cortical porosity-related bone loss and involves the use of ionizing radiation, which limits its widespread use [34,35].

Likewise, the characterization of the *in vivo* loading environment of bones also presents significant technical challenges to researchers and clinicians. Although direct measurement methods, such as strain-gauge extensometry, have long been applied [36,37,38] and are widely considered to be the ‘gold standard’ for the assessment of bone and joint loading during physical activity [39], biomechanical models have been most commonly used. Typically informed by measurements of ground reaction force, muscle activity, motion analysis, and increasingly by inertial measurement units [40], various models have been constructed to estimate potential forces applied to appendicular and axial bones during walking, running, and jumping, among other activities [41,42,43,44]. However, the process involves multiple stages, each with assumptions and errors that can cause a much larger compounded error [45]. As cautioned by Curry et al. [46], validation still represents a key limitation of many of the models that have been constructed. The advent of publicly available data sets, including direct measurements of joint contact force during walking (for example, [47,48]), has resulted in substantial improvement in the sophistication of modeling techniques [49], with the construction of patient-specific models, in particular, demonstrating encouraging accuracy for the estimation of the hip or tibiofemoral joint contact forces [50,51,52]. However, the extensive personalization of model parameters remains a time-consuming procedure and typically requires detailed medical imaging data that are not routinely available [53]. Thus, the capacity of validated musculoskeletal models to predict absolute contact forces during activities of daily living, other than walking-related tasks, has not been well studied [54,55].

### 2.2. Tendon

Several approaches for the non-invasive quantification of the mechanical properties of soft connective tissues, such as tendons, have been reported within the literature, including vibrational techniques [56,57,58], shear-wave elastography [59], and standard ultrasound imaging coupled with inverse dynamic models [60,61]. To date, most studies evaluating tendon properties have used standard B-mode ultrasonography coupled with inverse dynamics to indirectly estimate tendon displacement and loading commonly experienced during isometric muscle contractions (for example, [62,63]). The structural and material properties of the tendon, such as its stiffness, elastic modulus, and hysteresis, are then subsequently derived from either force–displacement or stress–strain curves. Although insightful, the indirect estimation of internal tendon properties using ultrasonography and inverse dynamics requires a number of key assumptions and has some significant limitations [64]. For instance, tendon loads derived from inverse approaches have been shown to be overestimated by as much as 50% compared to direct measurements [65,66], while estimates of viscous properties (hysteresis) have been shown to be physiologically implausible [67]. Indeed, direct measurements of the *in vivo* loading environment of human tendons have been made with relatively high accuracy via the use of implantable transducers, such as extensometer gauges, Hall-effect transducers, and fiber optic systems. The operating principles and limitations of each approach have been detailed elsewhere [68]. Although direct measurements of tendon force have been made during a number of activities of daily living, including walking, running, squatting, hopping, jumping, and cycling [69,70,71,72,73,74,75,76,77], these techniques are highly invasive, may interfere with normal movement patterns, often raise ethical concerns, and are commonly limited to small sample sizes. Moreover, to the best of our knowledge, they have not specifically evaluated mechanical loading in chronic tendon disease.

Shear-wave elastography, which has recently appeared in clinical settings, is among the better-established alternative modalities for quantifying the mechanical properties of muscles and tendons [78]. In contrast to standard ultrasound imaging and transmission-mode methods that use ultrasound compression waves, the technique estimates the material stiffness of tissues by measuring the velocity of shear waves rather than compression waves, which are typically generated either by the operator via the application of external forces or by an acoustic radiation force [79,80,81]. While elastography has been used for some time, with varying levels of success as a diagnostic aid in the assessment of chronic liver disease [81] and breast cancers [82], applications to musculoskeletal soft tissues are becoming increasingly popular [83,84,85]. By quantifying the velocity of the ultrasound shear wave, the technique has the potential to quantify the elastic modulus of tissues, which, subsequent to the non-linear properties of tendons at physiological loads [86,87], may also provide some insight into the internal loading environment of the tissue [59,88]. However, mathematical modeling studies have shown that longitudinal shear-wave velocity is unaffected by the tensile viscoelasticity of soft tissues [89,90], and thus, caution is required in its clinical use. Indeed, in a recent *ex vivo* study implementing both shear-wave and transmission-mode ultrasound methods, Glozman et al. [91] showed that estimates of longitudinal shear-wave velocity, although strongly affected by the initial mechanical state of the tissue, were confounded by tissue non-linearity. This finding has been corroborated by animal studies, which observed no correlation between shear-wave velocity and the tensile elastic modulus of healthy rabbit Achilles tendons [92]. Similarly, in a recent *in vivo* study comparing shear-wave elastography with standard B-mode ultrasound coupled with inverse dynamics, Misfud et al. [93] noted that estimates of the tendon modulus were only moderately correlated (r = 0.64) and only at low levels of tendon loading (10% maximum voluntary contraction). Although there is some evidence that longitudinal shear-wave velocity in tendons may be sensitive to its strain-energy dissipation properties [94], shear-wave measurements have also been shown to be highly sensitive to confounders, and thus, reliability and reproducibility are thought to be greatly limited [95,96,97].

### 2.3. Skeletal Muscle

The characterization of the mechanical properties of skeletal muscle at the organ level is complicated by the contractile nature of the tissue. Some approaches have been proposed to measure the passive mechanical properties of skeletal muscle; however, there is little consensus within the literature concerning the most appropriate measurement method. Indeed, in the context of sarcopenia, a syndrome characterized by an accelerated loss of skeletal muscle mass and strength [98,99], many modalities have been used to characterize the “quantity,” and to a lesser extent, the “quality,” of skeletal muscle, including anthropometry [100], bioelectrical impedance analysis [101], DXA [102], ultrasound imaging [103], magnetic resonance imaging [104], and radiographic computed tomography [105,106]. The advantages and limitations of each modality, as they relate to the measurement of muscle mass, have been summarized elsewhere [107,108,109]. Arguably, the current reference standards for quantifying the passive mechanical properties of skeletal muscle tissue include radiographic computed tomography (CT) and magnetic resonance (MR) imaging, which are capable of providing high-resolution cross-sectional images that allow the differentiation between skeletal muscle and other lean mass components [110,111,112]. Measurements of cross-sectional skeletal muscle area or volume, which are often allometrically scaled to measurements of body height, can be subsequently made to yield relative estimates of muscle mass, such as the skeletal muscle index (cm^2^/m^2^) [113]. Surrogate estimates of muscle composition and quality may also be obtained on the basis of signal intensity or the diffusion anisotropy of water in the case of MR imaging or with the transformation of attenuation coefficients to radio densities in the case of CT, which reportedly relate to muscle fat content [104,114]. MR imaging has also been applied in elastographic approaches as a measure of passive skeletal muscle properties [108]. As with ultrasound-based elastography, MR elastography relies on the transmission of an external vibration through skeletal muscle but uses MR imaging to measure the subsequent propagation of displacement waves through the tissue. Maps of shear modulus are then calculated based on the linear viscoelastic wave equation. A review of the technical details of the approach is provided elsewhere [115]. To date, the majority of MR elastography studies have assumed muscle to be isotropic [116,117,118]. Anisotropic approaches require additional information regarding muscle fascicle architecture, which can, for instance, be obtained from diffusion imaging [119,120]. Such methods have been used to study the elastic and viscous properties of both healthy and disease-affected muscles in their passive state [120,121,122]. While MR elastography is generally considered more reliable than ultrasound elastography [123,124,125], there is growing evidence that it may also be sensitive to contraction-induced stiffness changes, primarily at low levels of muscle contraction [116,122,126,127], which may contribute to lower apparent reliability [108]. Moreover, the typical scan duration and physical restrictions associated with MR elastography make it impractical to measure changes in the mechanical properties of skeletal muscle during “real-world” dynamic activities of daily living, such as walking, running, and stair climbing [127].

The force-generating capacity of skeletal muscle is most commonly estimated *in vivo* using simple dynamometry. Given the well-established relationship between muscle size and maximum voluntary force output [128,129], dynamometric measurements of skeletal muscle strength or torque are often normalized to image-based measurements of muscle volume or cross-sectional area to yield relative measurements of muscle strength. It is well known, however, that increases in strength do not necessarily correspond to increases in skeletal muscle mass [130] and that force generation in skeletal muscle is the result of a number of additional factors, including tissue composition (e.g., contractile versus non-contractile), architecture (intramuscular fiber orientation, length, and pennation), and neural activation (such as motor unit recruitment patterns, central drive, etc.), that contribute to the overall force production capabilities [131,132,133]. Hence, while electromyography has been used to estimate skeletal muscle force [134], electromyography-based estimates of muscle force can be substantially inaccurate [135]. Arguably, the estimation of the “specific tension” of a muscle (the maximal force per unit of physiological cross-sectional area) [136,137] may be a more appropriate indicator of the contractile quality of skeletal muscle. However, such estimates typically require the combined use of multiple modalities (dynamometry, ultrasound, MR, electrostimulation, and electromyography) to address issues related to the variation in motor unit recruitment, central drive, and participant motivation, to quantify muscle architecture, joint centers, and moment arms, and to address assumptions regarding agonist and antagonist muscle contributions to the net joint moment, each of which has errors that can result in considerable compounded errors [138] and that may partly underpin the wide variation in specific tension, for example, from 150 kPa [136] to 540 kPa [139], reported for human skeletal muscle measured *in vivo*.

## 3. Techniques for Quantitative Transmission-Mode Ultrasound

Although ultrasound compression waves have long been used for the non-destructive characterization of defects in engineering materials, first appearing in the 1930s [140], it was not applied in humans for the evaluation of musculoskeletal tissue properties until 1958, when it was first used, with limited success, to evaluate cortical bone status during fracture healing [141]. However, it was some 25 years later that the clinical utility of quantitative ultrasound was demonstrated with the publication of the seminal work of Langton et al. [142], in which transmission-mode ultrasound was used to assess cancellous bone.

In contrast to the qualitative information commonly afforded by the visual observation of ultrasound waveforms in A-mode, tomographic images in B-mode, and dynamic images in M-mode, quantitative transmission-mode ultrasound evaluates the fundamental properties of musculoskeletal tissue based on the interactions of propagating ultrasound compression waves with the tissue microstructure [143,144,145]. Indeed, the propagation characteristics of ultrasound compression waves through musculoskeletal tissues have been shown to be related to the mechanical properties (elastic coefficients) of the tissue, as well as to other characteristics, including mass density and macro- or microarchitecture, which, in turn, are associated with biomechanical properties [146,147,148,149,150]. Hence, quantitative ultrasound techniques, such as transmission-mode ultrasound, have been used to assess the material and structural properties of connective tissues and their change with loading, providing new insights into the health of musculoskeletal tissues and the biomechanical control of activities of daily living.

**Figure 1:**
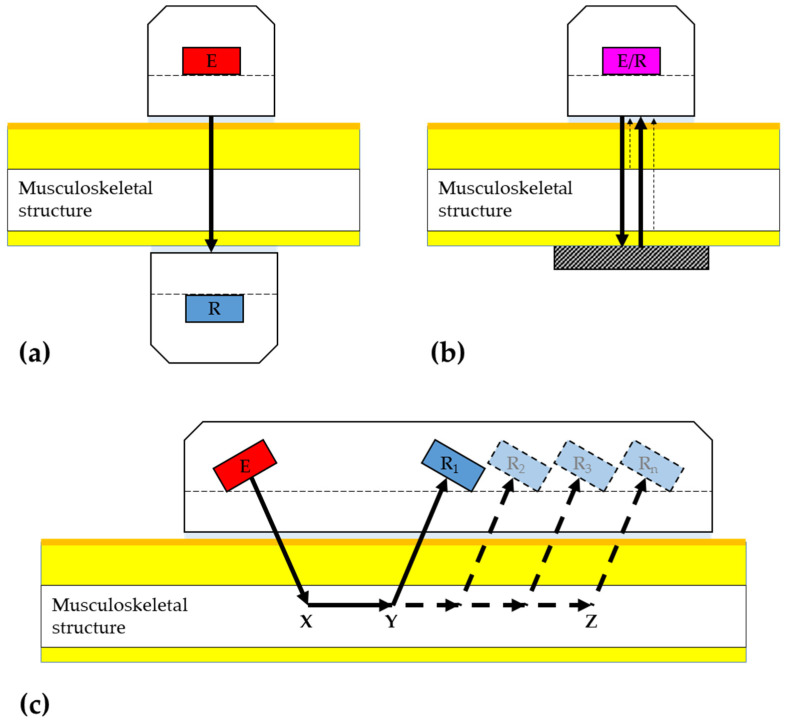
Illustration of the basic setup and operating principles of classical transmission-mode ultrasound techniques. (**a**) In the through-transmission technique, two ultrasound transducers, i.e., an emitter (E: red) and a receiver (R: blue), are placed on opposing sides of the musculoskeletal structure and provide estimates of tissue properties in the transverse plane. (**b**) Pulse-echo transmission approaches use a single ultrasound transceiver (E/R), operating in transmit and receive modes. Tissue characteristics are estimated by evaluating the backscattered signal and typically require assumed knowledge or combined measurements of one or several material properties. In soft tissues, the approach is often used in combination with a reflector (gray) positioned at a known distance from the transceiver. (**c**) Axial-transmission techniques typically employ two or more linearly orientated and regularly spaced ultrasound transducers consisting of an emitter (E: red) and one or more receivers (R: blue) aligned along the same side of the musculoskeletal structure. A broadband ultrasound pulse enters the musculoskeletal structure at a critical angle, and the compression wave propagates along the long axis of the structure.

Transmission-mode techniques using ultrasound compression waves may be generally classified into one of three categories (Figure 1): 1. through- or transverse-transmission, 2. pulse-echo, and 3. axial-transmission. While the current paper provides a brief overview of the operating principles behind each technique, a comprehensive discussion concerning the equipment, operating principles, analytic approaches, and limitations associated with each one and their many variants have been detailed elsewhere [151,152,153].

### 3.1. Through-Transmission Techniques

The through-transmission technique uses a separate emitter and a collinearly aligned receiver positioned on opposite sides of the musculoskeletal structure to be measured (Figure 1a). The distance between the emitter and receiver is either known or measured. Assuming that the system response and wave propagation are linear, the transmission characteristics, such as attenuation and velocity, of a broadband ultrasonic excitation transmitted through the sample can be readily obtained using the classical substitution technique, in which the signal transmitted through the musculoskeletal site is compared to that transmitted through a reference medium, such as water, at a known temperature [142,144,152].

### 3.2. Pulse-Echo Transmission Techniques

In contrast to through-transmission, pulse-echo transmission uses a single ultrasound transceiver, operating in both transmit and receive modes. Upon entering the skin and subcutaneous tissues, the broadband ultrasonic pulse is scattered and reflected from point-normal orientated interfaces where there is an acoustic mismatch between media, passes through mutual interference, and is received by the transceiver as radiofrequency echo signals (Figure 1b). Key characteristics of the target tissue, such as its thickness and attenuation, are subsequently estimated by evaluating the backscattered signal, which returns along the same path as the transmission [154,155,156]. In general, pulse-echo measurements of musculoskeletal tissues *in vivo* rely on assumed or a priori knowledge or combined measurements of one or several material properties, such as ultrasound velocity [153]. In some instances, an external reflector of a known distance from the transceiver is employed, thereby minimizing the need for prior knowledge [157,158].

### 3.3. Axial-Transmission Techniques

The axial-transmission technique, in contrast, uses two or more transducers, one acting as an emitter and the others as receivers [159]. The regularly spaced transducers are linearly orientated and axially aligned along the same side of the musculoskeletal structure to be measured (Figure 1c) [147]. Axial wave propagation differs considerably from conventional through-transmission and pulse-echo measurements. A broadband ultrasonic excitation is transmitted through the subcutaneous tissue and enters the musculoskeletal structure at a critical angle [160]. The refracted wavefront propagates along the longitudinal axis of the structure and, in doing so, results in a lateral wave that radiates into the subcutaneous tissue at a critical angle [161]. The transit time of the first arriving lateral wavefront along a defined distance is measured and used to calculate velocity. When applied to structures thicker than the involved wavelength, the apparent velocity of ultrasound in axial transmission corresponds to the speed of compressional bulk wave within the musculoskeletal structure [161,162]. The use of two or more receivers makes it possible to overcome the influence of the overlying skin and materials on the front of the probe when calculating wave speed [147,163]. As illustrated in Figure 1c and shown through Equations (1)–(6), the Time of Flight (ToF, in s), defined as the time the lateral wave of ultrasound pulse takes to travel along its transmission pathway, is measured at each receiver. The axial velocity is subsequently calculated by dividing the known distance between receivers by the ToF.

(1) $$ToF_{R_{1}-R_{n}}\left(s\right)=ER_{n}\;\left(s\right)-ER_{1}\;\left(s\right)$$

(2) $$\mathrm{where},\;ER_{1}\;\left(s\right)=EX\left(s\right)+XY\left(s\right)+YR_{1}\left(s\right)$$

(3) $$ER_{n}\;\left(s\right)=EX\left(s\right)+XZ\left(s\right)+ZR_{n}\left(s\right)$$

(4) $$\mathrm{as},\;YR_{1}\left(s\right)=ZR_{n}\left(s\right)$$

(5) $$ToF_{R_{1}-R_{n}}\left(s\right)=XZ\left(s\right)-XY\left(s\right)$$

(6) $$\mathrm{which}\;\mathrm{simplifies}\;\mathrm{to}\;\;To\;F_{R_{1}-R_{n}}\left(s\right)=YZ\left(s\right)$$

### 3.4. Key Transmission-Mode Measurement Parameters

The two most common parameters measured using transmission-mode ultrasound techniques are the propagation velocity (*V*, in m/s) and attenuation, reported either at a single frequency (α, in dB/m) or as frequency-dependent attenuation (FDA, in dB/MHz/m). From these measurements, a number of composite measurements have also been derived, particularly for bone, which have been reviewed elsewhere [164].

The longitudinal velocity of an ultrasound wave (*V*) in musculoskeletal tissues is dependent on the instantaneous elastic modulus (*E*) and mass density (*ρ*) of the tissue through which it propagates and is governed by the following relationship [143,149,165,166]:

(7) $$V=\sqrt{\frac{E}{\rho }}\;k$$

where k is a constant relating to Poisson’s effects and the negative ratio of axial to transversal strain with loading, which for connective tissues, such as tendons, is independent of the applied load [167]. Moreover, in soft connective tissues, which typically operate within the so-called ‘toe region’ under physiological loads, the axial velocity of ultrasound can be used as a surrogate measure of elastic modulus as it has been shown to vary monotonically with the applied load [148,149,168,169,170]. Hence, although the change in ultrasound velocity with loading represents a limitation of most sonographic approaches used to characterize soft-tissue properties [171], transmission techniques take advantage of this relationship, along with the non-linear properties of soft connective tissues at physiological loads [86,87], to also quantify the loading of soft tissues *in vivo* during dynamic activities of everyday living [172,173,174].

The propagation of ultrasound waves through tissue also results in a loss of signal intensity or attenuation via a number of processes, including both viscous and relaxation absorption [175], scattering, reflection [176], diffraction [177], mode conversion, and phase cancellation [152,178]. The loss of intensity or apparent attenuation (*α*) can be obtained from the referent (*A_ref_*) and measured sample (*A_sample_*) signals in either the time or frequency domain using the following equation [179]:

(8) $$\alpha =20\;\ln\frac{\left|A_{ref}\right|}{\left|A_{sample}\right|}$$

At the frequency range commonly used *in vivo*, ultrasound attenuation in musculoskeletal tissues typically varies quasi-linearly with frequency [180,181], with attenuation over the 0.2–0.6 MHz frequency range commonly referred to as broadband ultrasound attenuation (BUA) [144]. Table 1 shows the approximate ultrasound velocity, attenuation, tissue density, and elastic modulus values of muscle, tendon, and bone samples.

## 4. *In Vivo* Application of Transmission-Mode Ultrasound

### 4.1. Measurement of Bone Properties

All three ultrasound transmission techniques (i.e., through-transmission, pulse-echo, and axial-transmission techniques) have been used in the evaluation of passive properties of human bone, primarily within the context of osteoporosis and fracture risk. While each technique has been applied to a variety of skeletal sites, the increasing number of commercially available devices, which differ in terms of their measurement parameters coupled with the absence of technology-specific guidelines, has limited the widespread acceptance of transmission-mode ultrasound for the determination of passive bone properties for osteoporotic fracture risk within clinical settings [201]. The most common applications of each technique, however, are briefly reviewed below.

**Figure 2:**
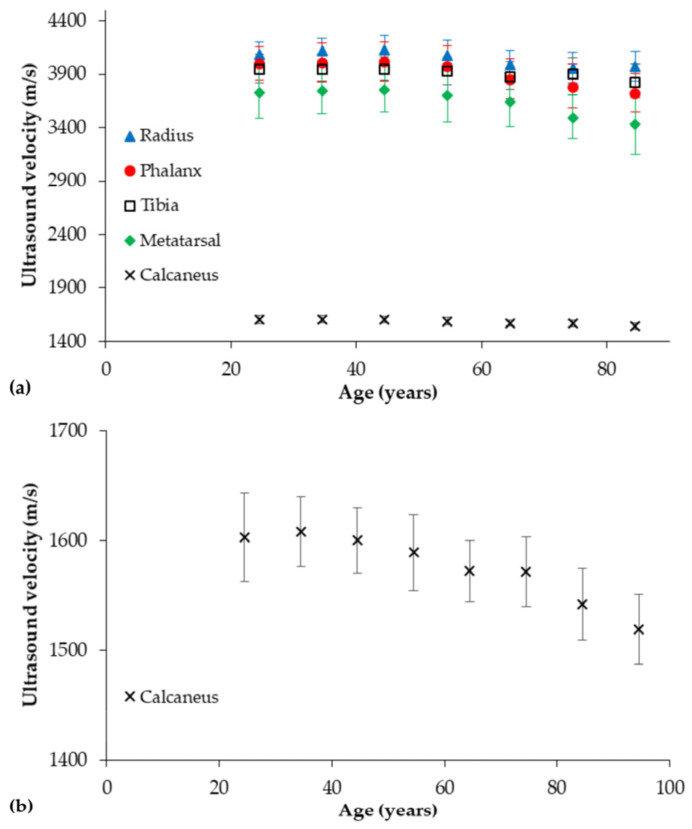

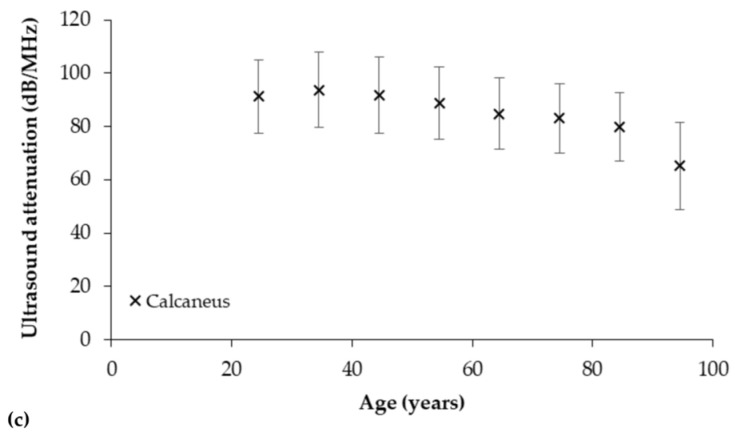
(**a**) Mean ultrasound velocity of the radius, phalanges, tibia, and calcaneus reported in healthy adults (n = 2969) [211,212,213,214,215]. Note that measurements of ultrasound velocity are site-specific. (**b**) Mean ultrasound velocity and (**c**) attenuation reported at the calcaneus as a function of chronological age in cross-sectional studies of healthy adults (n = 1836) [211,212,213]. The error bars depict the standard deviation.

The earliest and arguably best-validated approach in bone involves through-transmission measurements [201]. The majority of research employing the through-transmission technique has evaluated the calcaneus due to its high trabecular content, metabolic activity, and similar demineralization pattern to that of vertebrae [202,203]. However, the method has also been applied to evaluate the bone status of the proximal femur [204], patella, distal tibia, ulnar, radius, and phalanges [205,206,207,208], with the latter generally considered a measure of cortical properties (i.e., “cortical transverse transmission”) [209,210]. As shown in Figure 2a, measurements of ultrasound velocity are site-specific. In one of the earliest studies, Gerlanc et al. [208] reported ultrasound velocity through the distal tibia and ulna utilizing a through-transmission technique; the measurements were performed at regions of relatively constant and minimal soft-tissue thickness between the tibial tubercle and medial malleolus and between the olecranon and ulnar styloid, respectively. The ultrasound velocity values for both tibia and ulna in males demonstrated a rise from the third decade to the fourth decade, with a gradual decline thereafter. In contrast, females exhibited an increased ultrasound velocity at the tibia and ulna from the third decade to the fifth decade, followed by a greater decline in ultrasound velocity with age. The ultrasound velocity in people with an overt fracture at the tibia initially decreased but then increased non-linearly with healing. Delayed-union was characterized by a longer period of reduced ultrasound velocity before gradually increasing.

*Ex vivo* studies employing the through-transmission technique to evaluate trabecular bone have long since demonstrated that ultrasound velocity is closely related to its elastic modulus and, to a lesser extent, mass density [188,216]. Overall, similar age-related changes in ultrasound velocity profiles to those noted in the ulna and tibia [208] have been reported with trough-transmission measurements of the calcaneus in male and female populations (Figure 2b). In contrast to measurements of ultrasound velocity, measurements of ultrasound attenuation are thought to primarily reflect indices of bone mineral density and trabecular microarchitecture [217,218,219,220,221,222,223]. Langton et al. [142], in seminal work that greatly influenced later developments, applied the through-transmission technique to demonstrate, for the first time, that the slope of frequency-dependent attenuation of ultrasound at the calcaneus could be used to discriminate osteoporotic from non-osteoporotic individuals. Subsequent research demonstrated that, similar to measurements of velocity, ultrasound attenuation was sensitive to the effects of senescence, showing a reduction in attenuation values after the third decade (Figure 2c). Further research involving cross-sectional and longitudinal cohorts has provided considerable evidence that through-transmission measurements of ultrasound velocity and attenuation at the calcaneus are predictive of vertebral and femoral neck fractures in osteoporotic and several non-osteoporotic groups [224,225,226,227,228,229], with predictive rates comparable to or higher than that of radiographic-based measurements of bone mineral density [224,228,230,231,232]. They have also been shown to be predictive of low-trauma fractures in people with type 2 diabetes and chronic kidney disease [231,233], in which traditional X-ray-based clinical tools are generally considered insensitive [234].

A recent study of the UK Biobank, which includes over half a million adults recruited via the National Health Service, has also shown that calcaneal ultrasound velocity measured using the through-transmission technique is also independently associated with measurements of blood vessel compliance [235]. More recently, it was shown that broadband ultrasound attenuation of the calcaneus, but not apparent ultrasound velocity, was an independent predictor of both cardiovascular and all-cause mortality, even after adjustment for established cardiovascular risk factors and hip BMD [236], highlighting the potential utility of calcaneal ultrasound velocity to also evaluate the risk of cardiovascular disease.

Axial-transmission approaches have also been specifically advocated for use in osteoporosis [237] since approximately 80% of fragility fractures involve the appendicular skeletal sites comprised of large amounts of cortical rather than trabecular bone [27]. Cortical thinning and increased intra-cortical porosity are key factors underpinning non-vertebral fracture risk [238,239], and apparent ultrasound velocity in axial transmission is partly dependent on intra-cortical porosity, mineralization, and cortical bone thickness relative to its wavelength [161,240]. The technique has been most commonly applied to evaluate the cortical properties of long bones including the radius, tibia, metatarsus, and phalanges [240,241,242]. Although the level of evidence supporting the use of through-transmission ultrasound of the calcaneus for evaluating fracture risk is considerably greater than for other transmission modes, there is compelling evidence that axial-transmission parameters are also capable of discriminating between individuals with an osteoporotic fracture (in the hip, vertebrae, or other site) from age-matched healthy individuals [160,243,244,245,246,247]. Current evidence from prospective studies, however, is generally mixed [248,249,250,251], with a recent prospective study of children suggesting that through-transmission-derived measurements of calcaneal ultrasound attenuation may be superior to axial-transmission measurements of tibial velocity for monitoring bone densitometric change during puberty [252]. Although tibial ultrasound velocity has been shown to reflect the strength and elastic modulus of cortical bone [253] and is correlated with tibial bone mineral density, and, to a lesser extent, mineral density at other skeletal sites [237], it has also been shown to be a poor discriminator of osteoporotic fracture [254].

Axial-transmission ultrasound has also been applied as a quantitative method to evaluate fracture healing in long bones. Indeed, the technique was first developed in 1958 to study cortical bone status during fracture healing, albeit with limited success [141]. As described by Bossy et al. [162], the application of the axial-transmission technique to cortical bone yields both a fast-moving axial compression wave and slower “guided” waves. The latter waves arise from the reflection, mode conversion, and interference of longitudinal and shear waves within the cortical structure [241]. Animal, simulation, and clinical studies have demonstrated that axial-transmission parameters, such as the time of flight and propagation velocity of the first arriving ultrasound signal (i.e., fast wave), when measured across a fracture site, can be used as an indicator of bone fracture and healing [162,208,255,256,257], with propagation velocity, rather than attenuation, being arguably more sensitive to changes in callus mineralization and porosity during the regeneration process [258,259]. Similarly, simulation and *ex vivo* studies have also suggested that guided waves, which are generally dispersive and influenced by both the periosteal and endosteal bone surfaces, may be even more useful for evaluating oblique and transverse fractures in cortical bone [241,260,261,262,263] and may even have the potential to detect osseous micro-cracks [264]. The technique also allows waveguide characteristics of the bone, such as cortical thickness and/or porosity, to be estimated from dispersion curves by fitting a theoretical waveguide model [265,266,267,268]. Although preliminary applications *in vivo* show promise [269,270], soft tissue properties, multimode overlap, and the conversion of guided waves still present a significant challenge for the routine *in vivo* use of ultrasonic guided waves for the quantification of cortical bone fracture [271,272]. Moreover, further prospective research studies are needed to demonstrate the clinical potential of axial-transmission techniques for quantifying fracture characteristics in cortical bone and the subsequent healing stages.

In one of the earliest clinical studies, Craven et al. [273] reported ultrasound velocity in the cortical bone of the radius utilizing a pulse-echo technique, dividing bone thickness derived from plain radiographs by measured ultrasound transit time. A small cohort of nine adult males (23–33 years) exhibited larger cortical thickness and ultrasound velocities than 11 female adults aged between 49 and 71 years. In a subsequent paper [274], the elastic modulus was estimated as the square of ultrasound velocity multiplied by bone density, derived using both plain radiograph and photon absorptiometry data. The same research group also presented a conference abstract reporting a pulse-echo method to estimate ultrasound attenuation in cortical bone, derived from measurements of inner and outer cortices at two sites of different cortical thicknesses [275]. Given that cortical porosity and thickness are important determinants of the mechanical properties of the bone [276,277], pulse-echo methods have more recently been employed to estimate transverse ultrasound velocity, cortical thickness, and osteoporotic fracture risk. Primarily targeting cortical bone sites, including the distal radius and tibia, the simplest approach estimates cortical thickness using a single-element pulse-echo configuration to record the time lag between reflections from the periosteal and endosteal interfaces of the distal radius. The apparent cortical thickness is subsequently derived based on the assumptions of a normal-incidence homogenous material with perfectly flat interfaces and a priori knowledge of an invariant radial ultrasound velocity [155,278]. The technique also assumes that specular reflections from periosteal and endosteal cortical interfaces are stronger than backscattered ultrasound signals from cortical pores. Nonetheless, when combined with basic anthropometric data, multi-site pulse-echo thickness estimates were reported to have varied sensitivity (80–94%) and specificity (60–88%) in identifying hip osteoporosis compared to DXA but did not enhance the identification of radiographically confirmed fractures compared to estimates based on age and bone mineral density alone [279,280,281,282,283].

Ultrasound backscatter parameters, which can also be obtained from pulse-echo measurements, have also been applied to explore bone quality at both cortical- and trabecular-rich sites [284,285,286]. Backscatter occurs as the ultrasonic pulse interacts with the porous microstructure of bone, namely solid–fluid impedance mismatches, to provide frequency-dependent information on the composition (5 MHz), structure, and mechanical properties (1–3 MHz) of the bone [285,287,288,289]. Case-control studies have suggested that, compared to DXA, backscatter approaches applied *in vivo* to axial and appendicular reference sites can generally distinguish between elderly adults with and without osteoporosis, albeit with varied sensitivity (65–92%) and specificity (74–95%) [290,291,292,293,294,295]. Research has also demonstrated the utility of pulse-echo backscatter parameters in identifying fragility fractures [281,296,297], with several prospective studies reporting predictive metrics for fractures of the hip, spine, and “all” sites comparable to, or better than, DXA [298,299].

Although some pulse-echo backscatter approaches are reportedly limited by non-uniformities in the acoustic near-field [300], preliminary longitudinal studies suggest that backscatter parameters are also sufficiently sensitive to detect changes in cortical and trabecular bones following extended (90 days) bed rest [301], with a sensitivity comparable to that of axial-transmission techniques [302]. Although pulse-echo backscatter approaches also show promise for monitoring neonatal bone status [303,304], prospective research studies are needed to evaluate the sensitivity of pulse-echo techniques to quantify early skeletal changes with growth and metabolic disease and following therapeutic intervention.

### 4.2. Measurement of Bone Loading

The majority of research undertaken to date has applied transmission-mode ultrasound techniques to evaluate the passive material and structural properties of the bone. In one of the few studies evaluating the effect of mechanical loading on bone properties, Liu et al. [305] reported that through-transmission measurements of ultrasound attenuation at the calcaneus were significantly reduced with body-weight loading in a group of pre- and postmenopausal women (n = 16 and 45, respectively). While measurements of ultrasound velocity were also increased with loading, albeit to a lesser extent, the authors noted that the loading-induced reduction in ultrasound attenuation was greater in postmenopausal than premenopausal women. The authors suggested that greater ultrasound attenuation likely reflected changes in the trabecular microarchitecture with body-weight loading, highlighting the potential of the technique to evaluate the mechanical response of the bone to loading during activities of daily living. With the exception of heavily demineralized bone, however, mechanical testing of whole bone specimens *ex vivo* typically does not demonstrate an initial period of non-linear deformation with loading [306,307], the so-called “toe region”, which is characteristic of soft tissues and facilitates the use of the technique for quantifying the dynamic loading of soft tissues. Moreover, preliminary work from our laboratory has also highlighted that body-weight loading results in a change in the orientation of the calcaneal bone and differential movement of the overlaying soft tissues, resulting in small changes in the location of measurements [308]. Indeed, it is well known that the orientation of the bone and thickness of the overlaying soft tissue can significantly affect clinical measurements of broadband ultrasound attenuation of the calcaneus when using the through-transmission approach, albeit such effects are more pronounced for measurements of calcaneal ultrasound velocity [309,310,311,312,313]. Moreover, in contrast to ultrasound velocity, an established theoretical relationship linking ultrasound attenuation to the mechanical properties of the bone remains elusive [152,314].

### 4.3. Measurement of Tendon Properties

Although through-transmission techniques were applied as early as the 1990s to quantify the interaction and dispersive effects of the equine tendon on the propagation characteristics of ultrasound waves *ex vivo* [143,315], it was not until a decade later that axial-transmission techniques were first applied to evaluate human tendon biomechanics *in vivo* [148,172]. By measuring the transmission velocity of ultrasound compression waves along (as opposed to across) the tendon, axial-transmission ultrasound is particularly well suited to evaluating dynamic tendon biomechanics *in vivo* and has been successfully applied in a series of human cohort studies to evaluate tendon biomechanics during static and dynamic loading conditions [172,173,174,316,317,318], in tendon pathology [174,316,319], and common clinical approaches used to modify tendon loading [320,321,322,323], as outlined below.

#### 4.3.1. Tendon Biomechanics during Common Activities of Daily Living

**Figure 3:**
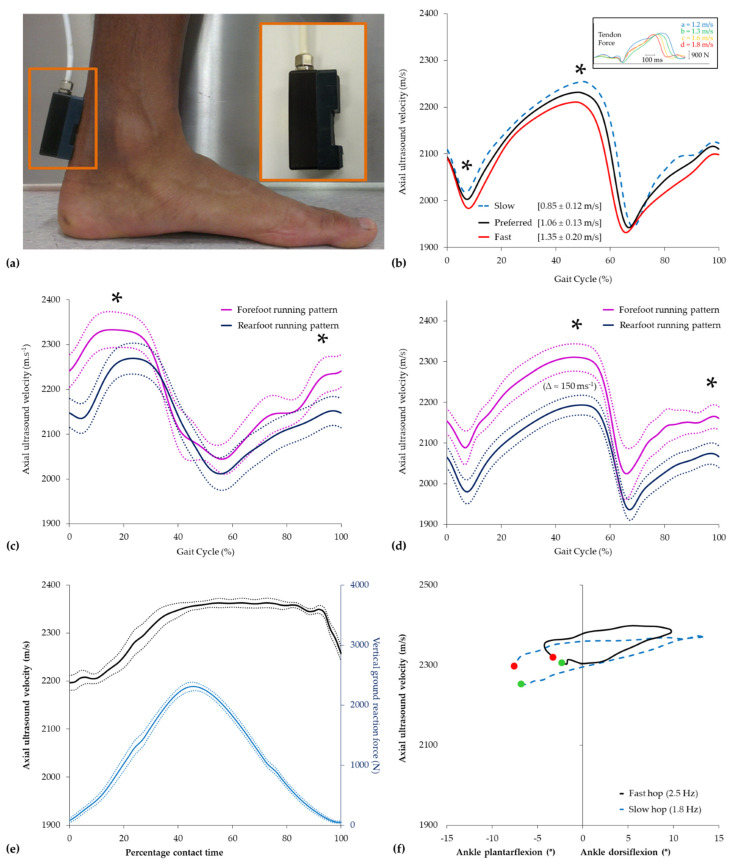
(**a**) Position of the axial-transmission probe over the Achilles tendon. (**b**) Consistent with research employing a direct measure of tensile force in the Achilles tendon (inset), axial velocity of ultrasound in the Achilles tendon is typically biphasic during walking, with peak values systematically reduced with increasing walking speed [173]. (**c**) Foot-strike patterns during running alter the axial velocity of ultrasound in the Achilles tendon [325]. (**d**) The effect of habitual foot-strike patterns on the axial velocity of ultrasound in the Achilles tendon during walking [325]. (**e**) The vertical ground reaction force (blue line) and axial velocity of ultrasound in the Achilles tendon (black line) during a 30-s hopping task [174]. (**f**) The effect of hopping at frequencies of 1.8 Hz (dashed line) and 2.5 Hz (solid line) on ankle movement and the axial velocity of ultrasound in the Achilles tendon [174]. The beginning of contact is denoted by a green circle (●), while the end of contact is denoted by a red circle (●). Note that the dotted lines represent standard deviations. The asterisks indicate a statistically significant difference (*p* < 0.05).

Axial-transmission techniques employing a nominal 1 MHz ultrasound emitter have typically been used to quantify the acute change in Achilles tendon (Figure 3a) and, to a lesser extent, patellar tendon properties during activities of daily living. During each heel–toe walking cycle, the axial ultrasound velocity trace in the Achilles tendon is characterized by two minima and two maxima (Figure 3b). The first minimum occurs shortly after the heel strike, at approximately 10% of the stance, following peak ankle plantarflexion, before peaking in the late stance at around 50% of the gait cycle shortly after peak vertical ground reaction force. The second and more profound minimum in ultrasound velocity occurs during the early swing phase at around 70% of the gait cycle before peaking again shortly prior to the next heel strike. The biphasic pattern was shown to be highly reproducible, with within-subject coefficients of variation typically <1.5% [317,320], and comparable to directly measured force profiles previously reported in the Achilles tendon with implanted force transducers [69,72,75]. While our unpublished data indicate that between-limb differences in axial velocity in the Achilles tendon during walking at preferred speeds are typically <1.5%, subsequent research has shown that increasing walking speed (0.85–1.35 m/s) resulted in a speed-dependent reduction in the axial velocity of ultrasound in the Achilles tendon during the stance phase of walking (Figure 3b), despite a concomitant increase in peak vertical ground reaction force and ankle plantarflexion [173]. The observation of a speed-dependent reduction in ultrasound velocity in the Achilles tendon is in close agreement with those in which force was directly measured within the tendon (Figure 3b inset) [72,75]. Although consistent with the limitations in the force–velocity behavior of the triceps surae muscle group [324], gait speeds associated with running were found to induce higher peak ultrasound velocities than for walking and, hence, higher internal loads within the Achilles tendon [317].

Subsequent research demonstrated that the foot-strike pattern adopted by runners had a profound effect on the axial ultrasound velocity recorded in the tendon during running (Figure 3c), with habitual forefoot-strike running patterns showing higher and earlier peak velocities than habitual rearfoot strikers [325]. The magnitude of the foot-strike effect (≈150 m/s) was marked compared to changes observed with walking speed but similar to that (≈140 m/s) observed with surgically induced injuries in the equine tendon [326]. Moreover, in comparison to habitual rearfoot strikers, runners that habitually adopted a forefoot-strike pattern also had markedly higher velocities in the tendon during walking (≈100 m/s) even though both groups adopted the same heel–toe walking pattern (Figure 3d). Hence, these findings highlighted that habitual footfall patterns during running may influence the functional properties of the Achilles tendon in recreational runners.

Hopping is a common activity, which is often used clinically to book-end progressive loading programs of the Achilles tendon during functional rehabilitation [327]. Direct measurements of physiological loading in the Achilles tendon have indicated that submaximal hopping places greater demands on the Achilles tendon than walking or running [74,75]. Research employing indirect estimates of tendon loading, in contrast, has suggested that Achilles tendon loads during bilateral submaximal hopping are akin to those of running [327]. To the best of the authors’ knowledge, only one study to date has applied transmission-mode ultrasound to quantify the change in Achilles tendon properties during hopping. Given that previous research has shown that the major determinant of leg stiffness during hopping switches from knee stiffness to ankle stiffness at a hopping frequency above 2.2 Hz [328,329,330], our laboratory recorded axial ultrasound velocity in the Achilles tendon of healthy adults while hopping at frequencies above (2.5 Hz) and below (1.8 Hz) this threshold [174]. At each frequency, ultrasound velocity profiles in the Achilles tendon were highly reproducible between cycles, with a mean within-subject coefficient of variation of <1% over the entire ground contact phase, and characterized by a single local minimum and maximum during the contact phase of steady-state hopping (Figure 3e). Maximum and minimum ultrasound velocities recorded in the Achilles tendon during steady-state hopping increased correspondingly with hopping speed (Figure 3f) and were approximately 200 m/s higher than those previously reported in healthy adults during walking at a preferred speed [148,172,320].

Although the peak axial ultrasound velocity in the tendon increased when hopping at the higher frequency, reflecting greater energy storage within the tendon, there was negligible change in the area of the hysteretic loop of the ultrasound-velocity–ankle-angle curve, a surrogate measure of energy loss of the tendon (Figure 3f). The results are thus consistent with *ex vivo* studies of tendon biomechanics in which viscous loss is known to be reduced in tendons exposed to higher strain rates [331]. Moreover, ultrasound velocity profiles in the Achilles tendon during hopping were found to be closely correlated with knee flexion (r = 0.95–1.00) rather than ankle movement (r = 0.35–0.79), highlighting the importance of knee movement to tendon loading and the challenge associated with the deconvolution of the contribution of agonist and antagonist muscles to the net joint moment, especially of bi-articular muscles encountered by inverse dynamic approaches [174].

**Figure 4:**
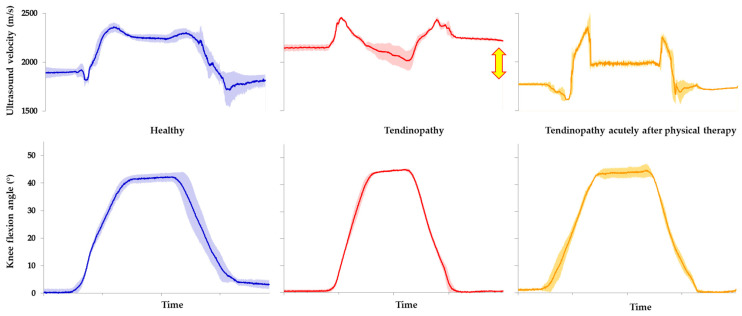
Ensemble axial ultrasound velocity traces in the patellar tendon (**upper panels**) and knee flexion (**lower traces**) during a squat movement in a healthy adult (**left panel**) and in an adult with unilateral patellar tendinopathy prior to (**middle panel**) [316] and immediately following a therapeutic intervention involving manual therapy (**right panel**). To aid in the comparison, traces are time normalized. Shading represents standard deviations determined over three movement cycles. Note the normalization of resting ultrasound velocity values in the painful tendon immediately after therapy (arrow).

Finally, axial-transmission ultrasound has also been used to measure *in vivo* properties and loading of the patellar tendon in adults during squatting, a common activity of daily living and exercise used in the rehabilitation of patellar tendinopathy. Axial ultrasound velocity in the patellar tendon was observed to increase non-linearly with knee flexion during the sit-to-stand movement in healthy adults; suggesting an increase in tension within the tendon, which peaked at around 15° of knee flexion [316] (Figure 4). Beyond approximately 20° of flexion, however, the ultrasound velocity within the patellar tendon remained relatively constant. The pattern of loading was highly reproducible (<3% within-subject coefficient of variation) but differed from that reported by previous research, in which inverse dynamics were used to estimate tension in the patellar tendon during squatting and peak force was estimated to occur beyond 80° of flexion [332]. However, the ultrasound velocity pattern in the patellar tendon was consistent with those of cadaveric research in which direct measurements of tendon loading showed that knee flexion beyond approximately 15° progressively lowered tension in the patellar tendon by as much as 50%, relative to that applied to the quadriceps tendon [333].

#### 4.3.2. Biomechanics of Injured Tendon

Tendon disease represents a spectrum of disorders ranging from reactive tendinopathy, through chronic tendinopathy, to rupture [334]. The most common clinical condition, chronic tendinopathy, is characterized by an underlying state of tissue degeneration [335,336], and is generally thought to reflect an “overuse” injury in which the tendon fails to adapt to prevailing loading conditions [337]. Ultimately, degenerative change associated with prolonged tendinopathy is thought to result in acute tendon rupture in selected cases, which often results in disability and long-term functional limitations [338].

To study the biomechanics of tendon injury, we used transmission-mode ultrasound to measure the *in vivo* loading of the patellar tendon in adults with and without unilateral tendinopathy during squatting [316]. Ultrasound velocity in the patellar tendon differed in young adults with and without chronic unilateral patellar tendinopathy (Figure 4). Despite tendinopathy involving only one tendon, ultrasound velocity was significantly higher (+250 m/s) in the tendons of both the symptomatic and asymptomatic limbs compared to that of healthy adults. The difference in velocity between groups was substantial, mirroring the magnitude of change reported in the equine tendon following surgery [326,339]. Moreover, the results were unexpected in two aspects. First, ultrasound velocity was higher bilaterally in injured adults despite the presence of only unilateral disease. We had hypothesized that ultrasound velocity would be lower in the involved tendon in tendinopathy, reflecting the disorganized collagen structure [335] and the inherently lower intrinsic elastic modulus of the tendon, as shown in previous studies in which an inverse dynamic approach was used to estimate patellar tendon stiffness in tendinopathy [340]. Second, ultrasound velocity was heightened in the tendon but only during a quiet bipedal stance both prior to and following the squat but not during the squat movement. We reasoned that if adults with tendinopathy possessed intrinsically stiffer tendons, which would account for a bilateral increase in ultrasound velocity, the axial velocity would be heightened throughout the entire movement, which was not the case. We concluded, therefore, that resting muscle tone may be increased in both asymptomatic and symptomatic tendons in patellar tendinopathy, resulting in greater loading and, hence, a higher ultrasonic velocity, bilaterally, while at rest [316]. Indeed, our findings were consistent with the so-called ‘vicious cycle’ pain theory and with previous research in which the electromyographic activity of agonist muscles is often higher in tendinopathy than in healthy controls [341]. Unpublished data from our laboratory have also highlighted the potential for axial-transmission methods to be used within clinical settings to monitor the effects of therapeutic interventions on the acute loading environment of the tendon (Figure 4).

**Figure 5:**
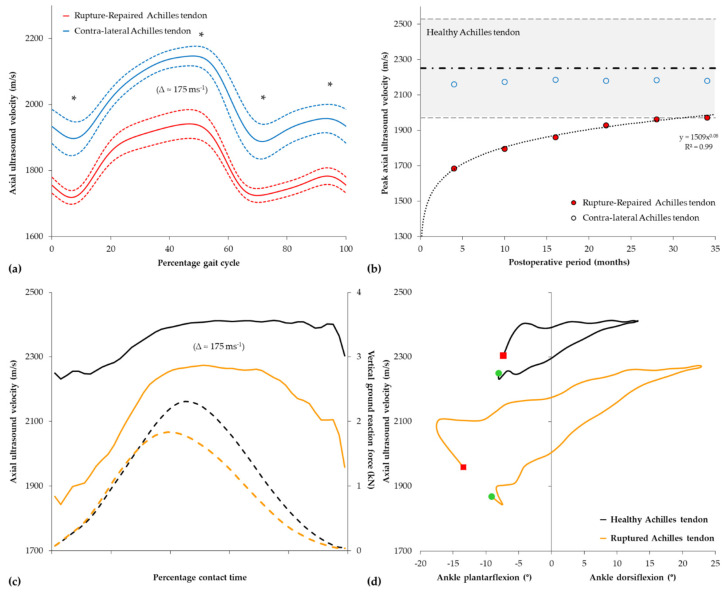
(**a**) Ensemble transmission speed of ultrasound profiles in surgically repaired (red) and contralateral (blue) Achilles tendons during barefoot treadmill walking at 1.05 m/s. Rupture-repaired tendons (n = 10) were characterized by a lower ultrasound velocity at all stages of the gait cycle [319]. The asterisks indicate a statistically significant difference (*p* < 0.05). The dotted lines represent standard deviations. (**b**) Repeated measurements of peak axial ultrasound velocity in the rupture-repaired (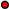: filled circle) and contralateral (**○**: empty circle) Achilles tendon of an adult male walking at a fixed speed (2.0 m/s), recorded at 6-month intervals over a 3-year post-operative period. Mean (dash–dot line) and 95% tolerance interval with 95% confidence (gray shaded area) for measurements of peak axial ultrasound velocity recorded in healthy adults (n = 50) walking at the same speed. Note the exponential recovery in the rupture-repaired tendon. (**c**) Axial ultrasound velocity (solid lines) and ground reaction force (dashed lines) profiles in surgically repaired (orange) and healthy (black) Achilles tendons during hopping at 150 bpm [174]. (**d**) Axial ultrasound velocity as a function of ankle joint angle in surgically repaired (orange) and healthy (black) Achilles tendons during hopping. The beginning of contact is denoted by a green circle (●), while the end of contact is denoted by a red square (■). Note that rupture-repaired Achilles tendon is characterized by a greater hysteretic loop than a healthy tendon.

Our laboratory has also used axial-transmission ultrasound to quantify the recovery state of rupture-repaired tendons. In a cross-sectional study of patients that had surgically repaired Achilles tendons, we observed that the axial velocity of ultrasound in rupture-repaired tendons was significantly lower than that of the contralateral limb during walking, despite symmetrical spatiotemporal gait parameters and vertical ground reaction forces between limbs (Figure 5a) [319]. The magnitude of the reduction in the ultrasound (≈175 m/s) was comparable to that previously reported in the equine flexor tendon (≈140 m/s) following the surgical induction of a core tendon lesion [326] and was suggestive of a lower material stiffness of the repaired tendon. The difference was even more pronounced during a hopping task (Figure 5c,d) [174]. We concluded that the lower material stiffness of the repaired tendon would alter the coordination and functional efficiency of the muscle–tendon unit, which may, in part, underpin reported tendon lengthening and long-term deficits in muscle strength following rupture repair [342,343]. Indeed, neuro-mechanical adaptations, consistent with our findings, have since been reported in subsequent research focused on chronic Achilles tendinopathy, the prequel state to rupture [344]. In contrast to hopping, we also observed no significant difference in the change in ultrasound velocity in repaired and contralateral tendons over a gait cycle. It is noteworthy, however, that the change in ultrasound velocity in the tendon during walking was strongly correlated with self-reported pain and physical function (r^2^ = 0.85, *p* < 0.01), as defined by the Achilles tendon Rupture Scale [345]. Unpublished data from our laboratory have also highlighted the potential for axial-transmission techniques to be used clinically to monitor the post-operative recovery of tendons following rupture. Figure 5b provides an illustrative example of recovery in the peak axial velocity of ultrasound in a surgically repaired Achilles tendon over a 3-year period following surgery in a male adult while walking on a treadmill at a fixed speed (2.0 m/s). Note that although the peak axial velocity in the rupture-repaired tendon increased exponentially throughout the post-operative period, to approach the lower 95% tolerance interval (with 95% confidence) of the healthy tendon (shaded area), it did not reach the peak axial velocity recorded in the contralateral tendon, which remained relatively unchanged over the 3-year post-operative period. Hence, transmission-mode ultrasound may have the potential to individually guide rehabilitation programs.

When considered collectively, these studies also highlight, for the first time, that different muscle–tendon units may respond differently to a tendon injury, resulting in either an increase or a decrease in tendon loading. Although neuromuscular mechanisms underpinning such a differential response are ongoing, from an applied perspective, such differences have important implications for tendon rehabilitation, in which progressive loading during activities of daily living remains the mainstay of treatment [327,346].

#### 4.3.3. Modification of Tendon Biomechanics via External Influences

Footwear remains a prime candidate for the prevention of tendinopathy and is often used as a key therapeutic intervention in Achilles tendon rehabilitation after injury. The inherent heel offset incorporated in traditional running shoes is thought to elevate the heel and shorten the muscle–tendon unit, thereby decreasing the load in the Achilles tendon during gait [347]. However, supporting evidence for such an effect is equivocal, with elevation of the heel in the order of 15 to 18 mm reported to either increase [348], decrease [349], or have no effect [350,351] on peak tensile loading of the Achilles tendon during running. In a series of repeated-measures studies, in which axial-transmission-mode ultrasound was used to monitor Achilles tendon biomechanics, our laboratory demonstrated that conventional running shoes that incorporated a 10-mm heel offset (elevation) significantly increased the peak axial velocity of ultrasound and hence, loading in the Achilles tendon during walking [320]. We later demonstrated that a simple orthotic heel lift (22-mm heel offset) may moderate the increase in loading to some extent [321] and that incorporation of a progressive increase in heel offset within the shoe (0 mm–15 mm) resulted in a linear reduction in the peak axial velocity of ultrasound in the tendon during walking [322]. However, we also observed that a substantial manipulation of footwear components, including heel offset, would be required to return the loading environment of the Achilles tendon to that of the barefoot gait [322]. More recently, we also applied this technique to demonstrate that the energy loss properties of shoes also alter the Achilles tendon loading during walking and may be manipulated to either increase energy storage within or reduce energy returned by the tendon [323].

### 4.4. Measurement of Skeletal Muscle

Quantitative ultrasound was first used *ex vivo* as early as 1982 to characterize the longitudinal and transverse changes in tissue stiffness with muscle contraction in animal models [352]. Using through- and pulse-echo transmission approaches (1, 3, and 7 MHz), Tamura and colleagues [352] demonstrated that, although the propagation speed of ultrasound waves in frog skeletal muscle (n = 20, mean (±SD) = 1610 ± 50 m/s) was virtually insensitive to passive force development, tetanic-induced isometric contraction resulted in an increase in propagation velocity in the longitudinal direction (≈245 m/s), coupled with a decrease in velocity in the transverse direction (≈283 m/s) [352,353]. Given that striated muscle stiffness has long been purported to reflect the number of active actin and myosin cross-bridges at any given time [354], Hatta et al. [353] used the same approach to argue that changes in axial ultrasound velocity, as surrogate measurements of muscle stiffness, and provided insight into the cross-bridge function in skeletal muscle.

Despite the pioneering work of Tamura and colleagues [352], the application of quantitative ultrasound techniques for the *in vivo* characterization of human skeletal muscle has been surprisingly sparse in comparison to that of the bone and tendon, only appearing within the literature relatively recently [158,355,356]. In contrast to the tendon, in which the axial-transmission technique predominates, research investigating changes in the properties of human skeletal muscle *in vivo* has almost exclusively adopted pulse-echo transmission techniques to measure the transverse velocity of ultrasound. In contrast to pulse-echo approaches used with the bone, measurements in muscles have most commonly employed a reflector, positioned at a known distance from the emitter [158,170,357,358]. While the use of a reflector aids in the accurate estimation of the transmission velocity of ultrasound waves, wavefront travel times have been shown to be highly sensitive to deviations in reflector inclination angles, which are likely unavoidable in clinical settings [359]. Nonetheless, the technique has been shown to be sufficiently sensitive (area under the receiver–operator characteristic curve = 0.94) to detect differences in the properties of the triceps surae muscles of young (mean (±SD) age: 28.1 ± 3.9 years) and elderly adults (82.7 ± 7 years), with the mean (±SD) transverse ultrasound velocity in young adults (1550 ± 8 m/s) reported to be significantly higher than that of the elderly adults (1523 ± 16 m/s) [158]. Clinical studies have also shown that, in comparison to ultrasound-based measurements of muscle thickness and echotexture, the technique was able to detect changes in skeletal muscle properties with short-term (7 days to 2 months) immobilization, with the transverse ultrasound velocity of the triceps surae muscle being significantly lower than that of the contralateral limb following immobilization [360]. Lower transverse ultrasound velocity, in turn, reportedly correlates moderately to the fat content of the muscle, as determined by the Dixon MRI fat fraction [361]. Consistent with measurements of through-transmission velocity in animal muscle [362], however, quantitative pulse-echo measurements of transverse ultrasound velocity in the muscle are reported to be relatively insensitive to the state of muscle contraction, with the isometric loading of the ankle (up to 650 N) shown to have a negligible effect on the ultrasound velocity (<10 m/s) of the calf muscle when measured in the transverse direction [360]. Hence, the pulse-echo technique, as currently used, does not appear suitable for characterizing the force-generating capacity or contractile quality of skeletal muscle.

**Figure 6:**
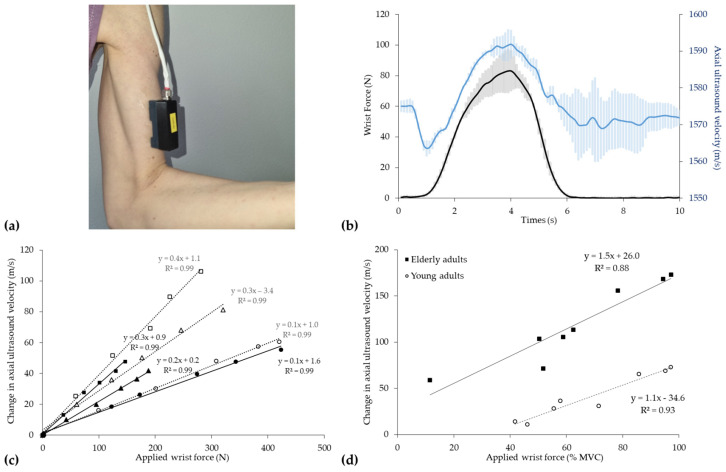
(**a**) Position of the axial-transmission probe (inset) over the belly of the biceps femoris muscle. (**b**) Ensemble force measured at the wrist (N, black line) and axial ultrasound velocity (m/s, blue line) recorded over the biceps femoris muscle during repeated maximum isometric voluntary contractions in a healthy male adult. (**c**) Change in peak axial ultrasound velocity recorded over the biceps femoris muscle in healthy individuals (n = 6) as a function of the force measured at the wrist during submaximal and maximal isometric contractions. Note that for each individual the relationship was best fit by a linear model; however, the slope varied markedly between individuals. (**d**) Change in peak axial ultrasound velocity recorded over the biceps femoris muscle as a function of the force measured at the wrist in young (n = 8 and mean age (±SD): 26.0 ± 6.5 years—empty circle) and elderly adults (n = 8 and mean age (±SD): 82.6 ± 6.4 years—filled square), expressed as a percentage of maximum voluntary isometric contraction. Note that for a given percentage of maximal contraction, elderly adults had a greater change in peak axial-transmission velocity from resting values than young adults.

The preliminary work from our laboratory suggests that axial-transmission techniques have the potential to provide suitable quantification of the contractile properties of skeletal muscle. Figure 6a demonstrates the measurements of axial-transmission velocity (nominal 1 MHz) recorded over the biceps brachialis muscle during maximum voluntary isometric contraction. Axial ultrasound velocity was monotonically related to the force measured at the wrist during isometric muscle contraction (Figure 6b), with the change in peak axial ultrasound velocity linearly related to force during submaximal and maximal isometric voluntary contractions (Figure 6c). It is noteworthy that, although the relationship was best fit by a linear model for each individual, the slope varied markedly among healthy adults. We also observed that for a given percentage of maximal isometric contraction of the biceps femoris muscle, elderly adults (n = 8 and mean age (±SD): 82.6 ± 6.4 years) demonstrated a greater change in peak axial-transmission velocity from resting values than young adults (n = 8 and mean age (±SD): 26.0 ± 6.5 years) (Figure 6d). Although further research is required, the results highlight, for the first time, the potential of axial-transmission techniques to quantify the contractile quality of skeletal muscle *in vivo*, particularly within the context of aging and age-related sarcopenia.

## 5. Future Perspectives

For many decades, researchers have worked to advance techniques used for characterizing musculoskeletal tissue properties *in vivo*. Considerable advances in transmission-mode ultrasound techniques have been made since the first *in vivo* application of the through-transmission approach at the calcaneus was detailed in the mid-1980s [142]. Technological developments, coupled with advances in data acquisition and signal processing procedures, have typically been adapted and applied to skeletal structures first, with the translation to soft tissue structures representing the next logical short-term development. Indeed, 3-D multi-frequency and multi-parameter measurements that combine transmission approaches are likely needed for the full characterization of the microstructural and mechanical properties of musculoskeletal tissues [363,364]. To that end, approaches such as ultrasound computed tomography (UCT) have the potential to effectively and efficiently quantify and spatially map human tissue properties in 3D [365,366], albeit at the expense of increased cost and complexity and reduced portability. Current applications have already demonstrated that UCT has the capacity to provide images from various reconstructed acoustic parameters, including the propagation velocity of axial ultrasound waves [367], density, and attenuation [358]. Sequential scanning approaches, such as those used in synthetic transmit aperture (STA) imaging [368], represent a natural extension of current bi-directional transmission approaches [270] and may be a useful adjuvant to aid multi-parameter imaging. Simultaneous developments in wearable ultrasonic arrays are likely to yield viable low-cost methods for extended, serial, and multi-parameter transmission measurements of musculoskeletal tissues that can be acquired at rest during activities of daily living and in settings outside the hospital or the laboratory [369]. Current wearable devices provide 48 h or more of continuous ultrasound scanning to depths of 30–40 mm, with contrast and axial/lateral resolutions in the order of ≈3 dB and 0.25/1.0 mm [370]. Such devices have already been applied *in vivo* to monitor skeletal muscle flexion [371] and the change in skeletal muscle modulus following exercise [372]. The future development of these approaches, and in particular, attenuation and backscatter parameters, would undoubtedly benefit from further elucidation of the complex interaction between ultrasound and the microstructures of connective tissues [152,179].

## 6. Conclusions

The assessment of the biomechanical properties of musculoskeletal tissues has long been recognized as an important tool in the evaluation of tissue adaptation, the early detection and management of pathological conditions, the staging of injury, and the monitoring of the progression of rehabilitation protocols. For this assessment, improving the accurate and non-invasive quantification of the *in vivo* properties of connective tissues in passive states and during activities of daily living remains a significant challenge. Although transmission-mode ultrasound techniques have been applied *in vivo* for the quantification of the structural and mechanical properties of human bone for over 40 years, these techniques have only been introduced relatively recently as non-invasive measurement tools for characterizing muscle and tendon biomechanics *in vivo*. The advancement of the techniques through continuing research will provide opportunities for new insights into the health and disease of musculoskeletal tissues and their biomechanical responses during activities of daily living.

## Figures and Tables

**Table 1:** Approximate values * reported for ultrasound velocity, attenuation, density, and elastic modulus of muscle, tendon, and bone.

Tissue	Velocity (m/s)	Attenuation (dB/MHz/cm)	Density (g/cm^3^)	Elastic Modulus (GPa)
**Bone**	2700–4100 [182,183]	2–15 ^†^ [182,183,184]	1.38–1.81 [182]	6.9–20.7 [46,185,186]
*Cortical*	2660–4200 [185,187,188]	5–12 ^‡^ [189]	1.66–2.10 [185,187,188]	10.9–19.7 [188]
*Cancellous*	1517–2892 [185,187,189,190]	4–31 [190]	1.08–1.76 [187,191]	3.0–15.0 [192]
**Tendon**	1637–1938 [143,168,187,193]	2–5 [184,193]	1.06–1.17 [149,168,187]	0.9–2.0 [60,194]
**Muscle**	1545–1631 [183,187,195,196,197,198]	0–3 [183,195,196,197,198,199]	1.04–1.18 [183,187,195,196]	0.1–0.4 [8,200]

* Approximate values vary with intrinsic, extrinsic and measurement-related factors including age, temperature and orientation. ^†^ dB/cm measured at 1 MHz. ^‡^ Radial measurement.

## Data Availability

No new data were created or analyzed in this study. Data sharing is not applicable to this article.
